# Echocardiographic findings in children with Marfan syndrome

**DOI:** 10.5830/CVJA-2010-085

**Published:** 2011-10

**Authors:** Osman Ozdemir, Rana Olgunturk, Serdar Kula, Fatma Sedef Tunaoglu

**Affiliations:** Kecioren Training and Research Hospital, Ankara, Turkey; Kecioren Training and Research Hospital, Ankara, Turkey; Kecioren Training and Research Hospital, Ankara, Turkey; Kecioren Training and Research Hospital, Ankara, Turkey

**Keywords:** dilatation of the aortic root, echocardiography, Marfan syndrome, mitral valve prolapse

## Abstract

**Background:**

The typical cardiac manifestations of Marfan syndrome are aortic regurgitation with progressive dilatation of the aortic root, which may cause dissection and rupture of the ascending aorta, mitral valve prolapse and mitral valve regurgitation. In this study, we aimed to show echocardiographic findings in 11 patients with Marfan syndrome.

**Methods:**

Diagnosis of Marfan syndrome was based on the Ghent criteria. All patients had a full echocardiographic evaluation. During the evaluation, we investigated the presence of mitral valve prolapse, mitral valve regurgitation, tricuspid valve prolapse, dilatation of the aortic root, and aortic regurgitation.

**Results:**

Eleven patients were diagnosed as Marfan syndrome (seven male, four female, age 4–14 years). All had mitral valve prolapse (nine with mitral valve regurgitation). Among these 11 patients, seven had accompanying tricuspid valve prolapse, six had dilatation of the aortic root and two had aortic regurgitation.

**Conclusion:**

Eleven patients in our clinic were diagnosed as Marfan syndrome since they had distinct characteristics of marfanoid phenotype. Echocardiographic evaluation of these patients showed marked heart valve involvement. In Marfan syndrome, it is known that the aortic valve is affected following mitral valve involvement. In our experience, aortic root dilatation is less common. However, particular attention should be given to following up aortic root status with non-invasive echocardiography to institute measures to prevent complications.

## Summary

Marfan syndrome (MFS) is an autosomal, dominantly inherited connective tissue disorder that affects the cardiovascular system, skeleton, eyes and lungs. Its incidence is two to three per 10 000 individuals.^1^-^3^ Mutation on chromosome 15 (15q21) and other mutations related to the fibrillin 1 (FBN1) gene have been reported.^4^,^5^ Despite these defined mutations, diagnosis of MFS is still based on clinical evaluation and family history.^1^ The Ghent criteria are used for the diagnosis.^3^ Typical characteristics of the disease are aortic valve regurgitation (AR) with progressive dilatation of the aorta, which may cause dissection and rupture of the ascending aorta, mitral valve prolapse (MVP), mitral regurgitation (MR), lens dislocation, myopia, thin stature with long extremities, arachnodactilia, chest wall abnormalities and scoliosis.^1^-^3^

Survival of the patients depends on preventing or controlling cardiovascular complications. Dilatation of the aorta is the main cause of mortality and morbidity in MFS, but it has been reported that MVP and MR are the most important causes of morbidity during childhood.^1^ In this study, echocardiographic evaluation of 11 children diagnosed as MFS during a four-year period is reported.

## Methods

In this study from January 2003 to January 2007, we report on echocardiographic findings of 11 patients who were evaluated for MFS in the Department of Paediatric Cardiology. The parents of all subjects signed an informed consent and the study complied with the Declaration of Helsinki and was approved by the local ethics committee.

Diagnosis of MFS was based on the Ghent criteria.^3^ In the absence of a family history of MFS, diagnosis was based on patients having major criteria in two different systems, with involvement in the third system, or having major criteria in one system, with involvement in the second system and mutation of FBN1. In patients with first-degree relatives with MFS, major findings in one system and involvement in the second system were used as diagnostic criteria.^1^-^3^

According to the Ghent criteria, major findings in the skeletal system are pectus carinatum, pectus excavatum requiring surgery, upper-to-lower segment ratio < 0.86 or arm span-to-height ratio > 1.05, wrist (Walker-Murdoch) and thumb (Steinberg) signs for arachnodactyly, scoliosis of > 20° or spondylolisthesis, reduced extension at the elbows (< 170°), medial displacement of the medial malleolus causing pes planus and protrusio acetabuli of any degree. Minor findings are: pectus excavatum, joint hypermobility, highly arched palate with crowding of teeth, and facial appearance (dolichocephaly, malar hypoplasia, enophthalmos, retrognathia, down-slanting palpebral fissures).

While ectopia lentis is a major finding in ocular evaluation, flat cornea, increased axial length of globe (< 23.5 mm) and hypoplastic iris or hypoplastic ciliary muscle causing decreased meiosis are minor findings.

Major findings in the cardiovascular system are dilatation of the aorta with or without AR, involving at least the sinuses of Valsalva and dissection of the ascending aorta. Minor findings in the cardiovascular system are MVP with or without MR, dilatation of the main pulmonary artery in the absence of valvular or peripheral pulmonary artery stenosis under the age of 40 years, calcification of the mitral annulus under the age of 40 years, and dilatation or dissection of the descending thoracic or abdominal aorta before age 50 years.

Lumbosacral dural ectasia on computerised tomography or magnetic resonance imaging is also a major finding. Other minor findings are spontaneous pneumothorax and apical blebs in the pulmonary system, striae atrophicae (stretch marks) that are not related to marked weight gain, pregnancy or repetitive stress, and recurrent or incisional hernia. The presence of a mutation that causes MFS in FBN1, a first-degree relative who independently meets the diagnostic criterion, and the presence of a haplotype around FBN1 inherited by descent and unequivocally associated with diagnosed MFS in the family are accepted as major findings in the family history.^1^-^3^

Using a commercially available echocardiographic system (General Electric Vivid 3 with 7- and 3-MHz probes), mitral and tricuspid valves were evaluated in particularly the parasternal long-axis and apical four-chamber views. Mitral valve prolapse and tricuspid valve prolapse (TVP) were defined as the presence of leaflet thickness > 5 mm and systolic prolapse of the leaflet(s) into the atrium for more than 2 mm.^6^

**Fig. 1:**
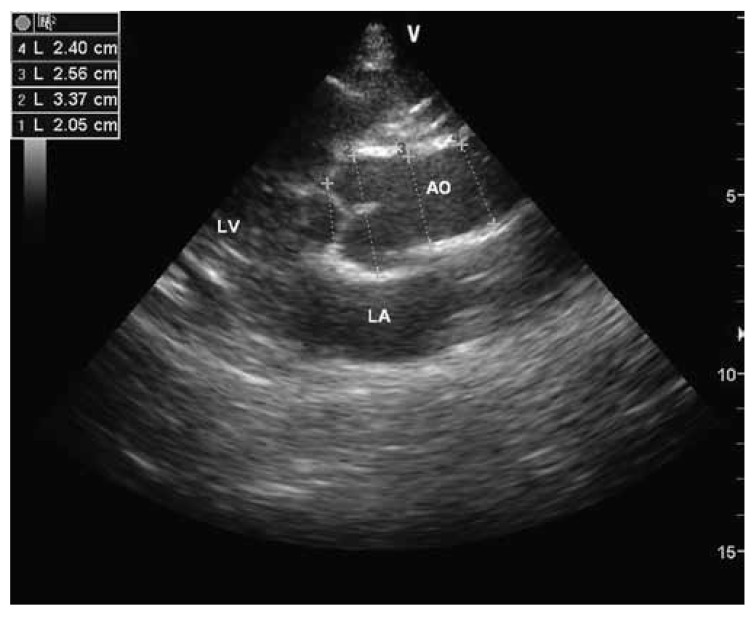
Aortic root measurements with cross-sectional echocardiographic examination. (1) annulus of the aorta, (2) sinuses of Valsalva, (3) supra-aortic ridge, (4) proximal ascending aorta. AO: aorta, LA: left atrium, LV : left ventricle.

During colour and pulse-wave Doppler assessment, > 1 cm colour regurgitation jet, peak flow velocity of the regurgitation flow > 2.5 m/s, and regurgitation flow during the systole or late systole were accepted as mitral regurgitation.^7^ Aortic regurgitation was defined as mosaic colour jet flow from the aortic valve to the left ventricle during diastole. Aortic root dimensions were measured at the level of the sinuses of Valsalva in M-mode and cross-sectional views (Fig. 1). Diagnosis from dilatation of the aortic root was based on the monogram described by Roman and colleagues.^8^

## Results

Eleven patients (four female and seven male, age range 4–15 years, median 11 years) presenting MFS phenotyping characteristics were diagnosed as MFS using the Ghent criteria.^3^ All patients had a physical examination and echocardiographic evaluation. Clinical characteristics of the patients are reported in Table 1.

**Table 1:** Patient Characteristics

*Patient no*	*Gender*	*Age (years)*	*BSA (m^2^)*	*MVP*	*MR*	*TVP*	*ARD (mm)*	*Limits of ARD based on BSA (mm)*	*DAR*
1	F	5	0.68	PAL	+	–	16	14–21	–
2	F	7	0.97	PBL	+	+	28	17–24	+
3	M	14	1.61	PBL	–	+	33	23–29	+
4	M	4	0.69	PAL	+	+	24	14–21	+
5	F	11	1.04	PBL	+	+	18	17–24	–
6	M	13	1.55	PBL	+	+	33	22–28	+
7	M	14	1.65	PBL	+	+	37	23–30	+
8	F	8	1.00	PBL	+	+	19	17–24	–
9	M	4	0.66	PBL	+	–	16	14–21	–
10	M	15	1.77	PBL	+	–	43	25–31	+
11	M	11	1.23	PAL	–	–	21	19–26	–

AR: aortic valve regurgitation; ARD: aortic root diameter; BSA: body surface area; DAR: dilatation of the aortic root; F: female; M: male; MFS: Marfan syndrome; MR: mitral regurgitation; MVP: mitral valve prolapse; PAL: prolapse of anterior leaflet; PBL: prolapse of bileaflet; TVP: tricuspid valve prolapse; +: present; –: absent.

The most notable clinical feature at the time of presentation was height equal to or greater than the 97th percentile. Arachnodactyly was found in all except one child. Nine patients had arm span-to-height ratio > 1.05. Eight subjects had a chest deformity. A pectus carinatum deformity was documented in five patients, while the excavatum deformity was noted in three subjects. A scoliosis was present in seven patients. Seven children had pes planus, which in most was associated with joint hypermobility. Highly arched palate with crowding of teeth was seen in 10 of 11 individuals. The classical facial features of MFS were noted in nine children. Ectopia lentis was found in only one subject. Three individuals had severe myopia (greater than 5 dioptres). A mutation in FBN1 was found in seven patients. No child experienced striae atrophicae, lumbosacral dural ectasia or spontaneous pneumothorax.

Regarding cardiovascular features of the patients, the ventricular functions were within normal limits. Mitral valve prolapse was documented in all patients, nine of whom had associated MR, whereas seven children had TVP. Eight patients had prolapse of both mitral valve leaflets while three of the patients with MVP had anterior leaflet prolapse. Dilatation of the aortic root was found in six patients (patient no 2, 3, 4, 6, 7 and 10). The range of the aortic root diameter was 16–43 mm and the median was 24 mm. The widest aortic root diameter recorded was 43 mm in a 15-year-old boy (10th case). None had an aortic dissection while two had AR. In the study, the fourth case (four years old and male) had all cardiac manifestations (MVP, MR, TVP, dilatation of the aortic root, and AR).

**Fig. 2:**
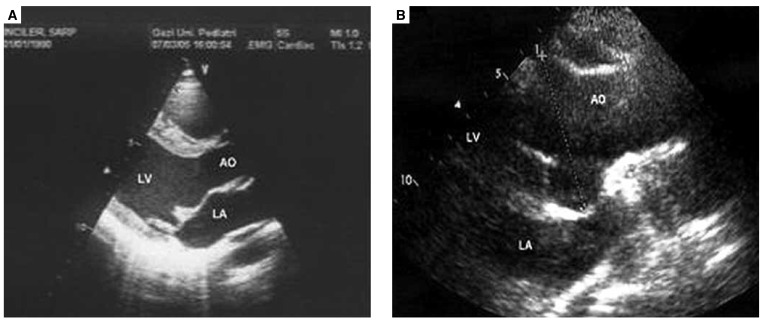
Mitral valve prolapse (A), and dilatation of the aortic root (B) in cross-sectional echocardiographic examination. AO: aorta, LA: left atrium, LV : left ventricle.

**Fig. 3:**
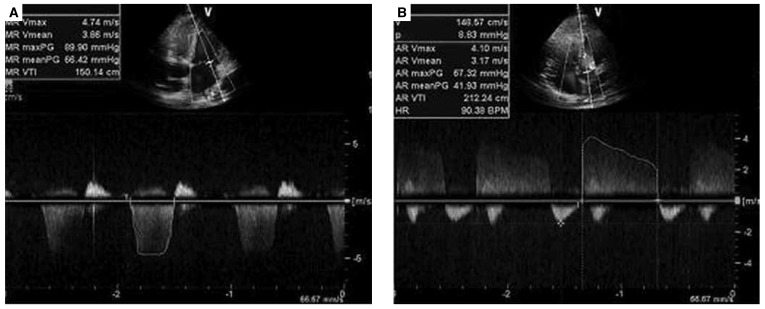
Mitral valve regurgitation in apical four-chamber view (A), and aortic regurgitation in apical five-chamber view (B) with pulse-wave Doppler.

Table 1 summarises the aortic root dimensions at the level of the sinuses of Valsalva, and the other findings of echocardiography. Figs 2 and 3 show examples of MVP, dilatation of the aortic root, MR and AR.

## Discussion

In MFS, cardiovascular disorders without appropriate treatment are the main cause of mortality in the first four decades of life. Extending survival depends on treatment or preventing complications of cardiovascular diseases such as dilatation of the aortic root, MVP, MR or dilatation of the pulmonary artery. Despite the diffuse abnormality of the aortic wall, enlargement usually occurs at the sinuses of Valsalva. This condition is seen in 70 to 80% of MFS patients and is more common in males. Dilatation of the aortic root usually begins in childhood. Independent of age, the rate of progression of the enlargement increases after the aortic diameter exceeds 5 cm. At this stage, the risk of dissection and rupture of the ascending aorta increases and therefore surgical intervention is suggested. Progressive dilatation of the aortic root also impairs leaflet coaptation and this causes AR.^1^–^3^

Mitral valve disease is present in 60 to 80% of the MFS population. This usually occurs in childhood before aortic involvement. Common mitral valve abnormalities are annular dilatation, fibromyxomatous changes in leaflet and chordae, elongated chordae, rupture of the leaflets and calcium deposition. Proximal pulmonary artery enlargement in the absence of pulmonary valve involvement or peripheral pulmonary artery stenosis, TVP, and coronary, axillary and subclavian artery aneurysms may also be seen in MFS.^1^-^3^

Among the patients evaluated for marfanoid phenotype, 11 were diagnosed as MFS. Although none had cardiac symptoms, dilatation of the aortic root was found in six patients and the diagnosis of MFS was based on cardiac involvement in this group of patients.^3^ Five others with valvular abnormalities without dilatation of the aortic root were diagnosed with the help of findings in other systems. These valvular abnormalities were accepted as minor findings, according to the Ghent criteria.^3^

Despite the fact that prognosis of patients with MFS depends on the presence of dilatation of the aortic root, there is insufficient information on aortic root measurements in children. There is consensus on measuring aortic root dimensions at the level of the sinuses of Valsalva but no agreement on which echocardiographic technique to use, cross-sectional or M-mode echocardiography. In our study, measurements were made from both cross-sectional and M-mode views and mean values of both measurements were used.^9^-^12^

Diagnosis from dilatation of the aortic root was based on the monogram defined by Roman and colleagues.^8^ Because aortic complications are more common in patients with a greater than 5% annual increase in aortic dimensions, serial aortic root measurements have prognostic importance in this patient population.^1^ Two of the patients diagnosed with dilatation of the aortic root had accompanying AR, which has no prognostic value in children because of its late occurrence.^10^

Mitral valve prolapse and MR occur before aortic valve involvement and show early progression in children with MFS.^1^ Consistent with this finding, MVP was detected in all our patients. In patients with MFS, unlike other causes of MVP, deformation of both mitral leaflets is more common and the need for surgical intervention is usually earlier.^1^-^3^

In our group, three patients had anterior leaflet prolapse while eight had prolapse of both mitral leaflets. Nine patients had marked MR and seven had TVP. Diagnosis of MVP and TVP was based not only on systolic prolapse of the leaflets but also thickness of the leaflets. Consequently, only classical MVP patients were assessed,^6^ however, marked mitral valve involvement and frequency of TVP was significant. Therefore, in patients with marfanoid phenotype, echocardiographic examination not only focuses on aortic root measurements, but must also include evaluation of the mitral and tricuspid valves from at least two different echocardiographic planes.

In our patients, systolic and diastolic ventricular functions were within normal limits. However, some studies have shown that even with apparently normal systolic or diastolic function using conventional echocardiographic examination, impairment of these functions can be detected with tissue Doppler imaging. These findings are explained as early manifestations of cardiac involvement.^13^ In addition, tissue Doppler imaging can be useful in evaluation of the aortic wall thickness.^14^ Therefore, supplementary use of tissue Doppler imaging in patients with MFS is important.

Cardiac symptoms in MFS are usually silent until adulthood.^1^ Although our patient group had marked cardiac involvement on echocardiographic evaluation, none had cardiac symptoms; therefore no therapeutic interventions were needed. However, early diagnosis of cardiac abnormalities and regular follow up of patients is important. Therapy with beta-blockers is suggested in these patients. With beta-blocker therapy, aortic stiffness is reduced and aortic distensibility increased. In young patient subgroups and patients with smaller aortic diameters, beta-blockers reduce the risk of sudden death and improve survival rates.^1^-^3^

## Conclusion

Even with no detected cardiac abnormality on first evaluation, echocardiographic follow up at six- or 12-month intervals is suggested, particularly in children with MFS.
